# Koch’s Tuberculosis Cure

**Published:** 1891-01

**Authors:** Herman Mynter


### KOCH'S TUBERCULOSIS CURE.

### LETTERS OF PROF. REISE, OF THE UNIVERSITY OF COPENHAGEN, GIVING THE BERLIN METHODS IN DETAIL.

(*From Hospitals Tidende.*)

TRANSLATED BY HERMAN MYNTER, M. D.

FIRST LETTER.

BERLIN, November 17, 1890.

I CONSIDER it proper to send a short report of what one sees in Berlin during these days. There are probably many who wish to hear something about the peculiar life which pulsates here on account of Koch's tubercular bacillus inoculations, Koch's lymph, and the results of it. Today I have for six hours continually examined patients, and if I now ask the question what seems sure and clearest in the proceedings of Koch, then the answer will be: Greatest and surest is the application of the method as a means of diagnosis. I have today seen a large number of patients in Bergmann's clinic, on which it is easy to see the correctness of the picture as Koch gives it, of tuberculous affections, which are external, and which you can see with your eyes. I saw patients with lupus, with the attached portion of the face covered with a crust, as for instance, an eruption of rupia, with swelling, heat and redness at the circumference, and at eight o'clock in the morning either suffering from severe fever or with a decreasing fever, which at 4 P. M. had completely disappeared. Some of these patients had been inoculated once, others twice, and one five times. The attack of fever which is produced by inoculation, consists of a quick and considerable rise of temperature, during which almost always a rigor occurs about three hours after inoculation. The rise of temperature reaches its maximum from six to sixteen hours after inoculation, and then decreases gradually simultaneously with the local symptoms which the inoculation has produced, that is, redness, swelling, etc., (inflammation of crust). The inoculation is repeated after the local and general symptoms have decreased, and the dose is increased if the last injection gives little or no reaction. About definite cure I shall give no opinion, having been here only one day, but those who have seen the patients for a longer time, insist that lupus can be perfectly cured. Only a short time is necessary to ascertain that all lupus tissue, even the smallest extension, becomes changed by inoculation in the way as Koch has described it. It is much more difficult during a short visit to find out its effect on tuberculous glandular swellings. The tissue is sure to swell and become tender after inoculation, and that an attack of fever after injection for lupus invariably develops. On the other hand, their decrease, as a result of the inoculation, not to say their perfect recovery, has, so far, no foundation, except what Koch says.

I have not seen any patient cured of this affection, which does not mean that I do not believe in it. The same may be said about numerous cases of tuberculous joint affections, of spina ventosa, and other affections of the bone. We see in these cases the inoculations being followed by local swelling and tenderness, and the characteristic fever, while both the local and general symptoms decrease in the same way as with lupus, but the final result and recovery can first occur after a long time, and, as far as I can see, it is a matter of faith rather than knowledge of the present time. I have today seen a case of coxitis, in which the fifth or sixth inoculation was made yesterday, and in which no reaction, local or general, had occurred, from which the conclusion was drawn that the tuberculous process had been destroyed and only the inflammatory process left, but "*nous verrons.*" While, therefore, the question about complete cure is still undecided, the same is not the case in regard to diagnosis. It must, after what I have seen, be considered proved that even tuberculosis in a human being who submits to Koch's inoculation is followed by both a local and general reaction. There may be, perhaps, a few cases on whom the inoculations have no effect, but, as a rule, reaction occurs by another injection of increased dose. The reaction is then a proof that a tuberculous affection is present, and it is impossible to obtain a better illustration than the one we had today in Bergmann's clinic. I examined with the laryngoscope five patients with laryngial affections: Number one, had a simple catarrhal swelling in the larynx; number two, a similar swelling on the posterior wall; number three, an ulcer on the posterior commissure; number four, an ulcer on the anterior commissure. All these four patients were supposed to be suffering from phthisis. Number five, had a small nodule in the larynx. All five were inoculated and the first four had the characteristic rise of temperature, going to 104 and 105°F., while the patient with cancer continued to have normal temperature. Therefore, the inoculation furnishes evidence of the presence or absence of tubercular bacillus, just as we saw it in the fifth case, that of cancer. After a number of inoculations in the same patient, the reaction decreases so that the second inoculation gives less reaction than the first, etc., when we increase the dose and wait for results. If no reaction occurs, the tuberculosis has been destroyed, according to the experience of the clinics in Berlin. It is only to be hoped that it is no immunity, but a final destruction of tuberculosis that is derived from repeated inoculations ; in regard to this, it rests for the future to show. While the dose in lupus is one centigram in adults, it is ten times weaker in tuberculosis of the lungs. In the same weak doses must be used in every case of joint or bone tuberculosis in which there is a suspicion that the lungs are affected, otherwise serious results may occur. I have seen little of the method for tuberculosis of the lungs, and it will probably take years before we arrive at a reliable opinion about its effect upon lung tissue, but that Koch's method will be an excellent diagnostic means for latent phthisis I have had the opportunity to verify. After what I have seen I would be careful to inoculate a phthisis patient having high fever, even with a weak injection of one milligram. Injection of a larger dose may produce a reaction lasting two, three, or four days, and may be longer ; vomiting and collapse during this period are not unknown. Bergmann showed me a patient with lupus, who had needed strong stimulation, in order to improve the imperceptible pulse, and to counteract the collapse. Is it not possible that this patient did have a latent phthisis ? I did not know, as I did not have the opportunity of examining him.

### SECOND LETTER.

While, in my first letter, I spoke particularly about lupus and tuberculous affections of glands, joints and bones, I will this time tell what I have seen and heard about phthisis pulmonalis. I have gained my experience particularly from the clinic of Träntzel. Koch's statement that the inoculations in phthisis pulmonalis must be much smaller, generally only one-tenth of the dose for non-phthisical patients, is confirmed everywhere. If—according to Koch—an inoculation of one cubic centimeter is used in a case of lupus, a dose of one cubic millimeter is the maximal doses in the beginning in case of phthisis pulmonalis. Even this dose can only be used in adults and comparatively strong patients. In children and very weak patients this dose must be considerably reduced, and if high fever be present it is prudent to commence with a dose of only one-half cubic millimeter, or even less.

The reaction of phthisical patients against the inoculation is quite considerable.

It is not only that the general reaction — the fever — and the local reaction which we suppose is present from the increased cough, increased expectoration and a considerable tension and pressure in the thorax is greater in patients who suffer from phthisis than in those who have only lupus or some external manifestation of tuberculosis, but phthisical patients do not seem to bear the inoculations as well as the other patients, as they can only stand one-tenth the dose, and besides are liable to get collapse.

Fatal cases, to be sure, have so far not occurred in the clinics I have visited, but some severe cases of collapse have occurred and that not alone in phthisical patients, but also in a patient with lupus. It is, therefore, necessary to use the inoculation with the greatest care and with proper regard to the patient's condition.

After inoculation on phthisical patients the usual reaction occurs in two, four or six hours; a rigor occurs, the temperature increases to 104 or 105, the local reaction shows itself by increased cough and expectoration, by tension and pressure in the thorax.

I have been unable to learn the influence of the local process upon the stethoscopical phenomena, but it is sure that the râles increase in number. The fever decreases in the course of ten to twelve hours, sometimes first after twenty-four hours. The local symptoms, such as increased cough, expectoration, and pressure in thorax decrease too, and the patients often feel improved. The patient's normal condition having returned, a new inoculation may be made, and if the reaction grows less and less with every new dose, it may be increased with one-half or one milligram, until by-and-by stronger doses are used,—even one centigram. One month will generally pass before such a dose can be used. In phthisical patients we see, then, a gradual decrease of the symptoms, diminished cough and expectoration, and the tuberculous bacilli decrease in number in the expectoration.

It is not an easy matter to treat a phthisical patient with Koch's lymph. The number of tuberculous bacilli in the expectoration must be counted before, during and after the reaction following the inoculation.

Already now I may state that none of our present hospital wards is able to treat a larger number of phthisical patients with Koch's method without an increase of the medical staff and of the number of nurses, if a scientific treatment, conducted with painstaking care, is demanded. It is probable that it will be necessary to open special wards in our hospitals for the treatment of a large number of phthisical patients, or rather to build new special hospitals.

The question, what will be the future result in phthisical patients and whether a perfect cure can be obtained, cannot be answered now, neither can I have any personal opinion about it. It will be necessary, first, to observe patients for months or half years.

Nothing is more common than for physicians to see phthisical patients recover so far that the pectoral symptoms, the cough and expectoration, disappear, the weight increase fifteen pounds or more, while at the same time the objective symptoms improve, the râles disappear, the dullness diminishes and grows less intense, the bronchial respiration stops. Yet we may quite unexpectedly see our patients get a new hemorrhage and new pectoral symptoms,—in short, get a relapse after months or years. And so it will go with Koch's method. Years will pass before we know its influence or the cause and progress of phthisis pulmonalis. We can only, till that time arrives, continue to work along the old line, making careful clinical observations, studying carefully the tuberculous bacilli, by aid of hygienic, dietetic and therapeutic measures, and by aid of the genial Dr. Koch's new remedy.

Koch's method seems least at place in extensively progressed cases of phthisis with high fever, and is here probably not without some danger. From what I have stated it is obvious that I have not seen a recovered case of phthisis pulmonalis in Berlin, but — my stay has been too short.

I accord great weight to the impression I have received by listening to, and talking with, eminent clinical teachers and most from Koch's reserved expressions. I believe I am able to state now that the results of Koch's method in phthisical patients must be investigated during a long course of time before we can draw final conclusions. Somebody must take the lead, and the somebody is necessarily Koch and Berlin. To conclude, my impressions are as follows :

1. Inoculation of Koch's lymph produces in tuberculous patients a certain and easily recognizable reaction, partly a general one in the form of a severe attack of fever, partly a local one, different according to the place of the tuberculous pains; externally or internally in the deeper organs — just as Koch so masterfully has described it. Two exceptions, perhaps, may be made; first, that it is not absolutely proved that a non-tuberculous patient never will show reaction even after a weak inoculation; and second, that perhaps as a great rarity reaction may not occur after inoculation in a phthisical patient. Certain doubtful cases are mentioned, but I have not seen them myself.

2. Koch's inoculations will be of the greatest importance as a diagnostic means of making us able to judge whether a given case is tuberculous or not.

3. It is too early to express an opinion about it as a curative measure. That it is curative in lupus is fully proved. It is probable that it will be found curative in other tuberculous affections too — according to the statement of Koch and others. I have only in these letters considered the clinical side of the question, leaving its pathological aspect till the remedy is known.

